# Exposure to second-hand smoke and direct healthcare costs in children – results from two German birth cohorts, GINIplus and LISAplus

**DOI:** 10.1186/1472-6963-12-344

**Published:** 2012-10-02

**Authors:** Ariane Batscheider, Sylwia Zakrzewska, Joachim Heinrich, Christina M Teuner, Petra Menn, Carl Peter Bauer, Ute Hoffmann, Sibylle Koletzko, Irina Lehmann, Olf Herbarth, Andrea von Berg, Dietrich Berdel, Ursula Krämer, Beate Schaaf, H-Erich Wichmann, Reiner Leidl

**Affiliations:** 1 Helmholtz Zentrum München, German Research Center for Environmental Health, Institute of Health Economics and Health Care Management, Member of the German Center for Lung Research, Neuherberg, Germany; 2 Ludwig-Maximilians-Universität München, Munich School of Management - Institute of Health Economics and Health Care Management and Munich Center of Health Sciences, Munich, Germany; 3 Helmholtz Zentrum München, German Research Center for Environmental Health, Institute of Epidemiology I, Member of the German Center for Lung Research, Neuherberg, Germany; 4 Technical University of Munich, Department for Paediatrics, Munich, Germany; 5 Ludwig-Maximilians- Universität München, Dr. von Haunersches Children’s Hospital, Munich, Germany; 6 UFZ - Helmholtz Centre for Environmental Research Leipzig, Germany, Department of Environmental Immunology, Leipzig, Germany; 7 University of Leipzig, Faculty of Medicine, Environmental Medicine and Hygiene, Leipzig, Germany; 8 Marien-Hospital Wesel, Department of Paediatrics, Wesel, Germany; 9 IUF Leibniz Research Institute for Environmental Medicine and Medical Faculty, University of Düsseldorf, Düsseldorf, Germany; 10 Medical Practice for Paediatrics, Bad Honnef, Germany; 11 Ludwig-Maximilians-Universität München, Institute of Medical Informatics, Biometry and Epidemiology, and Munich Center of Health Sciences, Munich, Germany

## Abstract

**Background:**

Although the negative health consequences of the exposure to second hand tobacco smoke during childhood are already known, evidence on the economic consequences is still rare. The aim of this study was to estimate excess healthcare costs of exposure to tobacco smoke in German children.

**Methods:**

The study is based on data from two birth cohort studies of 3,518 children aged 9-11 years with information on healthcare utilisation and tobacco smoke exposure: the GINIplus study (German Infant Study On The Influence Of Nutrition Intervention Plus Environmental And Genetic Influences On Allergy Development) and the LISAplus study (Influence of Life-Style Factors On The Development Of The Immune System And Allergies In East And West Germany Plus The Influence Of Traffic Emissions And Genetics). Direct medical costs were estimated using a bottom-up approach (base year 2007). We investigated the impact of tobacco smoke exposure in different environments on the main components of direct healthcare costs using descriptive analysis and a multivariate two-step regression analysis.

**Results:**

Descriptive analysis showed that average annual medical costs (physician visits, physical therapy and hospital treatment) were considerably higher for children exposed to second-hand tobacco smoke at home (indoors or on patio/balcony) compared with those who were not exposed. Regression analysis confirmed these descriptive trends: the odds of positive costs and the amount of total costs are significantly elevated for children exposed to tobacco smoke at home after adjusting for confounding variables. Combining the two steps of the regression model shows smoking attributable total costs per child exposed at home of €87 [10–165] (patio/balcony) and €144 [6–305] (indoors) compared to those with no exposure. Children not exposed at home but in other places showed only a small, but not significant, difference in total costs compared to those with no exposure.

**Conclusions:**

This study shows adverse economic consequences of second-hand smoke in children depending on proximity of exposure. Tobacco smoke exposure seems to affect healthcare utilisation in children who are not only exposed to smoke indoors but also if parents reported exclusively smoking on patio or balcony. Preventing children from exposure to second-hand tobacco smoke might thus be desirable not only from a health but also from an economic perspective.

## Background

In 2003, active smoking was estimated to result in 114,647 deaths in Germany, over 1.5 million years of life lost and 21 billion € of costs in terms of medical care and production losses [1]. The German Cancer Research Center estimated that in 2005 more than 8 million children in Germany lived in households with at least one smoker, and about two-thirds of all children aged 6-13 years [2], although the overall smoking prevalence in the German adult population was reported to be ‘only’ 29.9% in 2008/2009, depending on age, sex and level of education [3]. The detrimental consequences of second-hand smoke are already known [4-7]. In children, second-hand smoke can lead to respiratory diseases like chronic cough or asthma [8-11]. For children whose parents smoke, second-hand smoke was found to double the risk of otitis media [12] and increase the risk of sudden infant death syndrome two- to fourfold [13,14].

People exposed to second-hand smoke inhale the same damaging substances as active smokers. For children below the age of 3 years, exposure to tobacco smoke increases the amount of cotinine residues in urine, even if parents only smoke on balconies, patios and in gardens [15,16].

In addition to the health consequences of second-hand smoke exposure during childhood, a major concern is the related economic burden. International studies on the impact of second-hand smoke exposure during childhood on healthcare utilisation and costs are ambiguous. Previous studies have focused on different age groups, and used different methodologies concerning cost measurement and included components, as well as varying definitions of smoke exposure, so are not directly comparable. A Norwegian study reported a significant positive relationship between number of physician visits and smoke exposure in 15-year-olds [17]. The results of a US study in children under 12 years of age generally indicate a negative effect of a child’s exposure on the odds of any healthcare use but a positive effect on respiratory expenses as well as higher expenses among those with no respiratory expenses. However, the total effect of smoking at home on medical expenditures was not significant [18]. Other studies focused on costs related to respiratory diseases [19-21] or on younger children [19,22,23].

To date, German studies have focused on the behavioural and health consequences of second-hand smoke [24-29], whereas evidence regarding the economic impact of second-hand smoke in Germany is rare [30,31]. Using a top-down approach, Thyrian et al. estimate that more than 14,000 children are admitted to hospitals because their health is affected by exposure to tobacco smoke in their homes [30].

The aim of the present study was to estimate the excess healthcare costs of exposure to tobacco smoke over 12 months in children aged 9-11 based on two German birth cohort studies, GINIplus and LISAplus.

## Methods

### Study design and population

Our analysis is based on data from the GINIplus (German Infant Study On The Influence Of Nutrition Intervention Plus Environmental And Genetic Influences On Allergy Development) and LISAplus (Influence Of Life-Style Factors On The Development Of The Immune System And Allergies In East And West Germany Plus The Influence Of Traffic Emissions And Genetics) studies, two ongoing population-based German birth cohorts of healthy full-term neonates born between 1995 and 1999 in Munich, Wesel, Bad Honnef and Leipzig.

For the GINIplus study, 5,991 healthy full-term newborns were recruited from obstetric clinics in Munich and Wesel between September 1995 and July 1998 [32]. Details of the intervention and control groups can be found elsewhere [33]. In the 10-year follow-up, parents of 3,287 children completed questionnaires, which corresponds to a follow-up rate of 55% compared with the baseline survey [34]. The LISAplus study is an ongoing population-based birth cohort study of unselected newborns. A total of 3,097 healthy full-term newborns were recruited from 14 obstetric clinics in Munich, Leipzig, Wesel and Bad Honnef between November 1997 and January 1999 [35]. In the 10-year follow-up, 1,762 children participated in the study (follow-up rate compared with the baseline survey was 57%) [34]. Overall, we find for both studies that children staying in the cohort up to the 10-year follow-up have a higher socio-economic status at baseline compared to those dropping out at some stage. For both cohorts, approval was obtained from the respective local ethics committees (Bavarian General Medical Council, University of Leipzig, and Medical Council of North-Rhine-Westphalia), as well as written consent from the participants’ families.

Both studies had very similar study protocols, and shared identical standard operating procedures since the 6-year follow-up [32,35].

Combined, the GINIplus and LISAplus cohorts offer 10-year follow-up data on 5,049 children [34]. For 3,642 children (72% of the 10-year follow-up), information on healthcare utilisation is available. For 3,520 of these children, information on current smoking-status is available. Two children were excluded from analysis as they were identified as highly influential due to extremely high utilization. This exclusion was confirmed by the Walsh test and by standardised DFBETA values after a model including these observations was fit. This resulted in an analysis population of 3,518 children.

### Definition of smoking status

Parents were asked if someone had smoked in their homes during the last 12 months. Possible answer categories were “daily or almost daily”, “at least once per week”, “occasionally” and “never”. For those answering “never”, we asked if someone had smoked on the balcony or patio of their home or outside their home environment. With this information, tobacco smoke exposure was classified into four categories: “at home (indoors)”, “at home (patio/balcony)”, “not at home, but at other places” and “no exposure”. As information on the extent of smoke exposure was rather limited in the data, it was not taken into account.

### Covariates

We obtained information from both studies on socio-demographic characteristics from self-administered questionnaires filled in by parents. We used information on parents’ education as a measure of the socio-economic background of the children. Parental education is defined as maximum completed years of schooling of either of the parents, categorised as low (<10 years), medium (=10 years) and high (>10 years). Where information on parental education was missing (in 0.4% of mothers and 2.0% of fathers), we imputed the missing values with single imputation using the Markov Chain Monte Carlo (MCMC) method in SAS (PROC MI). We then used the completed education levels to impute missing income levels (9.8%) using the logistic regression method within PROC MI.

### Measurement and assessment of cost components

Direct medical costs were estimated using a bottom-up approach, i.e. based on patient-level data on several categories of utilisation of healthcare services. Parents were asked to state number of physician visits (for nine types of specialists), (physical) therapies (for seven types of specialists) and number of hospital days required by their child during the previous 12 months. Prices were based on a national costing guideline from the Working Group Methods in Health Economic Evaluation (AG MEG) [36], where available, and on information from relevant organisations as well as a survey of market prices. All prices were denominated in Euros and adjusted to the year 2007 following the AG MEG recommendations. A more detailed description on the methodology of monetary valuation is provided elsewhere [34]. As there was no information available on the reason for hospitalisation, we assessed the costs of inpatient hospital treatment by multiplying the number of days of the child’s stay by the mean costs per hospital day.

In the rare cases where information on type of healthcare service and frequency of utilisation was missing, we applied a three-step approach of missing value imputation: first, if parents did not state whether or not a specific type of service (physician/therapist/hospital) was used, we imputed the use of a service (yes or no) with single imputation using the MCMC method in SAS (PROC MI). The information was missing in 16 (0.5%) cases for physician visits, in 22 (0.6%) cases for therapist visits and in 19 (0.5%) cases for hospital stays. Second, we used the resulting information to impute whether a certain kind of specialist had been used (for physicians and therapists only), again with single imputation using the MCMC method. Third, we used the regression method in PROC MI to impute the number of specialist visits and hospital nights. We decided to use this imputation approach, as in a complete case analysis we would lose information for a total of 445 (12.6%) children, where in many cases only information on some cost category was incomplete. In particular, in the case of hospital utilisation, where information on utilisation was almost complete, complete case analysis would result in a disproportionate loss of information and an imprecise estimation of average utilisation and costs. In addition, cases with missing information were not distributed evenly between homes with and without smoke exposure. In a sensitivity analysis using only complete cases in the respective category, we found that results barely changed.

Healthcare costs were assessed for all children and compared between the different smoke exposure groups. As the correlation between health problems and second hand smoke exposure is not trivial and still not completely understood, this excess cost approach is the best way to capture all the differences between the analysed groups.

### Statistical analysis

To account for non-normality of the cost data, we estimated 95% confidence intervals (95% CIs) for differences between groups, applying a non-parametric bootstrap approach using the percentile method [37,38].

We analysed the effect of tobacco smoke exposure on direct medical costs, composed of physician, therapist and hospital costs, using a two-step approach, as a large proportion of the children incurred no costs. These zero-inflated data require an adequate statistical approach: first, we used a logistic model (LOGISTIC procedure in SAS) for the binary response variable to assess the odds of generating costs in the respective category. Second, we examined factors influencing the amount of costs using a generalised linear model (GENMOD procedure in SAS) assuming an inverse Gaussian distribution of the response variable and a log-link function [39,40]. In both steps, we controlled for sex, study centre, parental education, household poverty status and single parenthood. Recycled predictions were used to assess the overall association between smoking exposure and costs as well as the total cost differences between groups (smoking attributable total costs) across both steps of the two-part model by combining the effect on the probability of having positive costs and on the amount of costs given costs are greater than zero [41]. 95% confidence intervals (CI) for the adjusted cost differences were estimated from 1000 bootstrap replications using the percentile method.

P-values of ≤5% were regarded as statistically significant. Statistical analyses were performed with SAS software (SAS Institute Inc., Cary, NC, USA, version 9.2).

## Results

The socio-demographic characteristics of the study sample are presented in Table 1. In total, 55.2% of the children were exposed to second hand tobacco smoke at home (indoors or on patio/balcony).

**Table 1:** Description of the analysis population

**Characteristic**	**Classification/annotation**	**GINIplus and LISAplus study population analysed (n = 3,518)**	
		**N**	**Mean (SD) or (%)**
Age	Mean (SD)	3,518	10.1 (0.20)
Sex	Boys	1,802	(51.2%)
	Girls	1,716	(48.8%)
Study region	Munich	1,796	(51.1%)
	Wesel	1,162	(33.0%)
	Bad Honnef	190	(5.4%)
	Leipzig	370	(10.5%)
Net household income (€)	Up to 1,500	250	(7.1%)
	1,500 - < 2,500	891	(25.3%)
	2,500 - < 3,500	1,064	(30.2%)
	3,500 and above	1,313	(37.3%)
Poverty status of household relative to median equivalence income in Germany 2007	Up to 60% of median income	555	(15.8%)
	60-100% of median income	1,246	(35.4%)
	More than 100% of median income	1,717	(48.8%)
Education of the mother	Low (<10 years)	326	(9.3%)
	Medium (=10 years)	1,389	(39.5%)
	High (>10 years)	1,803	(51.3%)
Education of the father	Low (<10 years)	635	(18.1%)
	Medium (=10 years)	837	(23.8%)
	High (>10 years)	2,046	(58.2%)
Education of the parents	Low (<10 years)	181	(5.1%)
	Medium (=10 years)	943	(26.8%)
	High (>10 years)	2,394	(68.1%)
Single parent	No	3,142	(89.3%)
	Yes	376	(10.7%)
Child’s exposure to tobacco smoke	No exposure	752	(21.4%)
	Not at home, but at other places	825	(23.5%)
	At home (patio/balcony)	1,340	(38.1%)
	At home (indoors)	601	(17.1%)

SD: standard deviation.

### Utilisation and base analysis of costs

Table 2 shows the number of children with service utilisation, and unadjusted mean use of physicians, therapists and hospitals for 12 months.

**Table 2:** Utilisation of healthcare services over 12 months

	**Subjects using resources (%)**		**Mean frequency of utilisation (if used)**	**Standard deviation**	Unit cost per visit/day (€)*
Physician visits (total)	2,979	(84.7%)	4.4	4.2	
Paediatrician	1,955	(55.6%)	2.6	2.4	22.53
General practitioner	895	(25.4%)	2.2	1.6	18.87
Ophthalmologist	1,050	(29.9%)	1.4	0.9	30.80
Orthopaedist	538	(15.3%)	1.7	1.2	27.59
Ear, nose and throat specialist (ENT)	409	(11.6%)	1.9	1.7	29.29
Dermatologist	431	(12.3%)	2.2	3.2	18.40
Pulmonologist	101	(2.9%)	2.8	3.4	44.53
Other physician	346	(9.8%)	3.0	4.4	37.09
Emergency room	474	(13.5%)	1.3	0.7	44.62
Therapy (total)	891	(25.3%)	13.8	19.8	
Alternative practitioner	217	(6.2%)	4.2	10.6	22.00
Physical therapy	196	(5.6%)	14.1	16.1	20.18
Speech therapy	145	(4.1%)	16.8	14.6	22.58
Psychotherapy	183	(5.2%)	12.7	15.4	55.94
Occupational therapy	98	(2.8%)	21.6	17.1	26.58
Homoeopathy	207	(5.9%)	3.0	2.6	First: 86.56;
					Subsequent: 43.28
Other therapies	99	(2.8%)	11.8	15.3	31.33
Hospital (days)	193	(5.5%)	5.1	6.6	488.37

N = 3,518.

* Unit costs according to a national costing guideline [36] updated to the base year 2007.

Average annual medical costs of physician visits, physical therapy and hospital treatment were estimated to be €343 (313–380) per child. Costs were €286 (238–336) for children having no exposure to smoke, €312 (250–386) for children not exposed to smoke at home but at other places, €346 (302–394) for children in homes where smoking took only place on patio/balcony and €449 (330–603) for children exposed to smoking at home (indoors).

Descriptive analysis showed that exposure to smoke is associated with a higher total of physician, therapist and hospital costs (p < 0.001), with the largest share of these excess costs being caused by higher hospital costs.

### Regression analysis

First, we analysed the impact of exposure to tobacco smoke on the odds of incurring healthcare costs in the fields of physician, therapist and hospital use. The results are shown in Table 3. Smoke exposure at home, both indoors and on patios/balconies, resulted in higher odds of physician use or any of the considered services (p < 0.05). The impact of parental education and household’s relative income position, as well as sex of the child, on the odds of using healthcare services was limited. Children of families with single parents used therapists more likely (p < 0.001).

**Table 3:** Odds of using medical services

**Parameter**	**Physician use**	**Therapist use**	**Hospital use**	**Physician, therapist or hospital utilisation**
	Odds [95% CI]	Odds [95% CI]	Odds [95% CI]	Odds [95% CI]
**Intercept**	4.77 [3.78–6.02]	0.36 [0.29–0.43]	0.04 [0.03–0.07]	5.90 [4.60–7.56]
**Sex**				
Reference: Female	1.00	1.00	1.00	1.00
Male	1.03 [0.85–1.23]	1.16 [0.99–1.35]	0.92 [0.69–1.24]	1.08 [0.89–1.31]
**Study centre**				
Ref: Munich	1.00	1.00	1.00	1.00
Leipzig	1.59 [1.09–2.30]*	0.80 [0.62–1.04]	1.48 [0.93–2.34]	1.37 [0.94–2.02]
Bad Honnef	1.12 [0.72–1.75]	0.67 [0.47–0.96]*	1.11 [0.58–2.12]	1.01 [0.63–1.60]
Wesel	0.70 [0.56–0.87]**	0.52 [0.42–0.63]***	0.99 [0.69–1.43]	0.63 [0.50–0.79]***
**Parental education**				
Ref: High (>10 years)	1.00	1.00	1.00	1.00
Low (<10 years)	1.30 [0.82–2.05]	0.69 [0.45–1.06]	1.16 [0.60–2.24]	1.24 [0.77–1.98]
Medium (=10 years)	1.14 [0.91–1.44]	1.05 [0.86–1.27]	1.08 [0.75–1.55]	1.12 [0.88–1.43]
**Relative income position**				
Ref: > 100% of median income	1.00	1.00	1.00	1.00
0-60% of median income	0.88 [0.65–1.20]	0.87 [0.66–1.14]	1.19 [0.76–1.88]	0.90 [0.65–1.25]
60-100% of median income	0.82 [0.65–1.02]	1.07 [0.89–1.29]	0.81 [0.56–1.16]	0.79 [0.62–0.99]*
**Single parenthood**				
Ref: No	1.00	1.00	1.00	1.00
Yes	1.11 [0.80–1.54]	1.53 [1.20–1.96]***	1.01 [0.63–1.62]	1.11 [0.79–1.57]
**Exposure to tobacco smoke**				
Ref: No exposure	1.00	1.00	1.00	1.00
Not at home, but at other places	1.27 [0.97–1.66]	0.98 [0.78–1.23]	1.25 [0.77–2.01]	1.26 [0.95–1.68]
At home (patio/balcony)	1.37 [1.07–1.75]*	1.13 [0.91–1.39]	1.52 [0.99–2.33]	1.35 [1.04–1.75]*
At home (indoors)	1.51 [1.11–2.06]**	1.06 [0.82–1.38]	1.60 [0.96–2.64]	1.48 [1.07–2.04]*

N = 3,518.

Ref: Reference category.

Dependent variable: use of physicians/therapists/hospital/use of physicians, therapists and/or hospitals.

Assumptions: binomial distribution of the error terms, logit-link function.

***/**/*, significant at the 0.1%/1%/5% level.

In a second step, we considered the influence of exposure to tobacco smoke and confounding variables on the amount of costs if the respective services were used. Results are shown in Table 4. Evidence regarding the effect of exposure to tobacco smoke on healthcare costs is mixed: whereas physician and therapist costs are not influenced by exposure to tobacco smoke, hospital and total costs were significantly higher (p < 0.05). Compared with those not exposed, children who are exposed to smoke inside their homes incurred significantly higher hospital and total costs (p < 0.01), whereas for children in homes where smoking took place on patios or balconies, only the sum of all cost categories was significantly elevated (p < 0.05). As for the first step of the model, a child’s sex, parental education and the household’s relative income position did not play an important role regarding the amount of healthcare costs. Children of single parents incurred significantly higher therapist, hospital and total costs.

**Table 4:** Amount of costs when medical services are used

**Parameter**	**Exp(Estimate) [95% CI]**			
	**Physician costs**	**Therapist costs**	**Hospital costs**	**Sum of costs (physician, therapist, hospital)**
	**(N = 2,973)**	**(N = 880)**	**(N = 193)**	**(N = 3,038)**
**Intercept**	108.98 [99.10–119.83]	394.76 [295.91–526.63]	1778.92 [1298.02–2437.99]	302.84 [252.57–363.13]
**Sex**				
Ref: Female	1.00	1.00	1.00	1.00
Male	1.05 [0.98–1.13]	1.26 [1.01–1.57]*	0.98 [0.78–1.24]	1.11 [0.95–1.29]
**Study centre**				
Ref: Munich	1.00	1.00	1.00	1.00
Leipzig	1.06 [0.94–1.20]	0.76 [0.53–1.09]	1.21 [0.81–1.81]	1.26 [0.93–1.69]
Bad Honnef	0.99 [0.85–1.16]	0.78 [0.46–1.33]	0.91 [0.54–1.54]	0.82 [0.59–1.14]
Wesel	0.94 [0.86–1.03]	0.69 [0.52–0.91]**	0.96 [0.72–1.29]	0.71 [0.59–0.85]***
**Parental education**				
Ref: High (>10 years)	1.00	1.00	1.00	1.00
Low (<10 years)	1.06 [0.90–1.26]	1.19 [0.60–2.35]	0.86 [0.51–1.45]	0.88 [0.63–1.23]
Medium (=10 years)	1.08 [0.99–1.18]	1.13 [0.85–1.50]	0.89 [0.67–1.18]	1.10 [0.91–1.33]
**Relative income position**				
Ref: > 100% of median income	1.00	1.00	1.00	1.00
0-60% of median income	1.04 [0.92–1.17]	1.45 [0.92–2.29]	0.79 [0.56–1.11]	1.09 [0.86–1.39]
60-100% of median income	0.99 [0.91–1.08]	1.12 [0.87–1.43]	1.18 [0.87–1.61]	1.11 [0.94–1.33]
**Single parenthood**				
Ref: No	1.00	1.00	1.00	1.00
Yes	1.11 [0.98–1.25]	1.55 [1.03–2.33]*	1.57 [1.02–2.43]*	1.61 [1.18–2.20]**
**Exposure to tobacco smoke**				
Ref: No exposure	1.00	1.00	1.00	1.00
Not at home, but at other places	0.97 [0.87–1.08]	0.70 [0.50–0.97]*	1.33 [0.93–1.89]	1.02 [0.83–1.25]
At home (patio/balcony)	1.03 [0.93–1.14]	0.88 [0.65–1.20]	1.22 [0.88–1.71]	1.26 [1.02–1.54]*
At home (indoors)	0.92 [0.81–1.03]	1.00 [0.66–1.52]	1.83 [1.19–2.81]**	1.45 [1.11–1.89]**

Ref: Reference category.

Dependent variable: physician/therapist/hospital/sum of physician, therapist and hospital costs where respective costs >0.

Assumptions: inverse Gaussian distribution of the error terms, log-link function.

***/**/*, significant at the 0.1%/1%/5% level.

In summary, the odds of incurring healthcare costs by children in homes where smoking was reported either inside the home or on patios/balconies compared with children not exposed to smoking was increased by 48% and 35%, respectively. For those who incurred any healthcare costs, costs were higher by 45% and 26% respectively.

**Figure 1:**
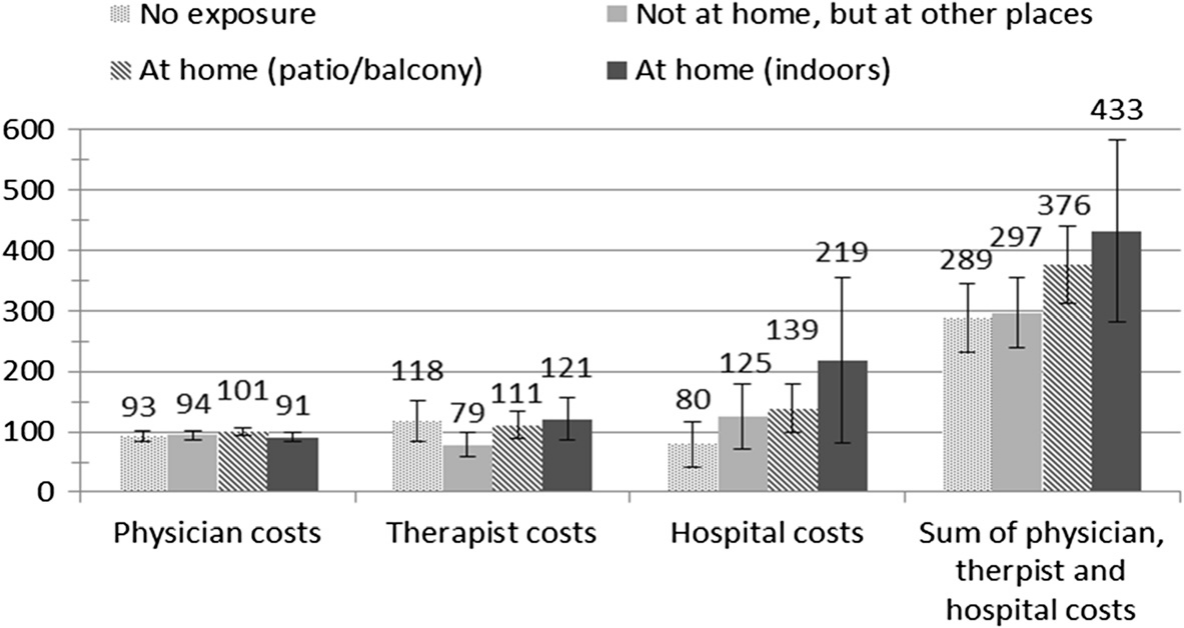
Predicted average adjusted costs by exposure to tobacco smoke.

In a third step, we combined step 1 (use/non use) and step 2 (costs of users), which allows for estimation of adjusted mean costs for the whole sample. Figure 1 shows these predicted mean costs for the single categories by second-hand smoking status. Predicted total costs are higher for the two groups of children with in-home exposure to second-hand smoke (patio/balcony and indoors) relative to those with either no in-home exposure (no exposure or not at home, but at other places).

Table 5 displays the resulting adjusted smoking attributable total costs per child with 95% confidence intervals. It shows that the differences for the two in-home exposure groups compared to “no exposure” are significant: Children whose parents reported smoking at home on the patio or balcony had excess costs of 87€ and children whose parents reported smoking at home indoors even 144€ compared to children who were not exposed to second hand tobacco smoke. The difference between the two in-home exposure groups (inside or on patios and balconies) is meaningful in magnitude (57€) but not statistically significant.

**Table 5:** Predicted smoking attributable total costs per child

	**Mean difference**	**[95% CI]**	**p-value**
No exposure	-		
Not at home, but at other places	8€	[−67€ - 76€]	0.824
At home (patio/balcony)	87€	[10€ - 165€]	0.030
At home (indoors)	144€	[6€ - 305€]	0.034

Means, confidence intervals (CI) and p-values were derived from 1000 bootstrap replications of the recycled predictions using the percentile method. Means correspond to differences in Figure 1.

## Discussion

This study investigated the differences in healthcare costs for children exposed to different modes of tobacco smoke using a bottom-up approach based on two German birth cohort studies. We found that children living in homes where smoking was reported (either inside or on patios and balconies) showed significantly higher medical costs than children not exposed to smoke. For children who are not exposed at home, but at other places, unadjusted medical costs were also slightly higher; however, these differences are not significant. These descriptive findings were confirmed by regression analysis. Thus, exposure to tobacco smoke is shown to have an effect on direct healthcare costs independent from socio-economic status. As the difference between the two in-home exposure groups (inside or on patios and balconies) is not statistically significant, attempts by parents who smoke to protect their children by smoking only on balconies or in gardens appear to be ineffective.

Measuring the burden of second-hand smoke in terms of implied healthcare costs rather than in terms of induced incidence or prevalence of diseases, has the advantage that impacts can easily be aggregated across different diseases. To indicate an impact from exposure to tobacco smoking, healthcare costs have to differ significantly, which is a more conservative approach than relying on a diagnostic label alone. Given that active smoking significantly increases the risk of age-related diseases such as heart and cardiovascular disease or cancer, one cannot expect comparable cost impacts of second-hand smoke on children with a mean age of 10 years. As the correlation between health problems and second hand smoke exposure in childhood is not trivial and still not completely understood, the excess cost approach is a possibility to capture all the differences between the analysed groups, but it does not allow for interpretation of causal pathways. The excess cost approach used in this study is aimed at identifying all excess healthcare utilisation due to either the condition under research itself (in this case smoke exposure) or any other disorder related to this condition, i.e. its consequences on health status. Thus, estimating the excess costs of exposure to second hand tobacco smoke in a data set comprising current utilisation of health care could potentially permit one to assess the total impact of this exposure on costs of care.

In addition to these strengths there are several limitations to this study. Both datasets provide information on healthcare utilisation for the 10-year follow-up. Unfortunately, it was only possible to include about 70% of the 10-year study participants due to missing data on healthcare utilisation or exposure to tobacco smoke. Like other retrospective cost assessments, this study was prone to recall error regarding utilisation of healthcare services. As far as recall abilities of parents are not associated with their child’s exposure to tobacco smoke, this should only influence the estimated average costs, not the differences between groups [42,43]. Additionally, our results may not be fully representative for the German population with regard to health-related as well as socio-demographic characteristics. The fact that families participating in the two birth cohorts show above-average education and income levels [34] may lead to an underestimation of actual costs, but should not influence cost differences substantially between the different groups of exposure to tobacco smoke. In addition, all study regions in this study are relatively urban. As in more urban areas, the supply of healthcare may be more extensive, this may lead to an overestimation of the actual healthcare costs. However, the comparison with the probability of physician visits in the representative KiGGS-Study [44] shows a similar picture for most comparable specialist groups.

Furthermore, the magnitude of healthcare costs related to second hand smoke exposure might be underestimated due to the design of this study. Since children with low birth weight, defined as birth weight below 2500 g, were not included in this study and low birth weight might be a result of maternal smoking during pregnancy, this study might have indirectly excluded some children exposed to tobacco smoke in utero which were shown to have an increased risk of hospital admission as infants in an earlier study by Adams et al. [45]. Moreover, it might be more likely that these children are also exposed after birth. Moreover, the excess cost approach may overstate the actual impact of parental smoking if there is unmeasured confounding that is associated with both, parental smoking and parental investment in child health endowments, e.g. a healthy diet.

Although the overall smoking prevalence in the German adult population was 29.9% in 2008/2009 [3], a previous study found that about two-thirds of all children aged 6–13 years were reported to live in a household with at least one smoker [2]. One reason for this gap might be that the smoking prevalence is higher in younger adults (e.g., 37.9% in 18–29 year olds) [3]. Furthermore, this gap might be explained by a possible higher concentration of larger families in lower-education smokers, as higher smoking prevalence was reported in people with lower levels of school education [3]. However, in our study the percentage of children exposed to tobacco smoke at home (indoors or on patio/balcony) was closer to one half (55%).

As about 45% of children included in the cohorts at birth did not participate in the 10-year follow-up, we are unable to rule out non-response bias. The proportion of people with high education was lower and the proportion of mothers smoking during pregnancy was higher in study dropouts than in participants. In addition, the proportion of children being exposed to smoke during their first year of life was higher in children who dropped out. As information on healthcare use and exposure to smoke was not available for all children at 10-year follow-up, we performed additional non-response analysis comparing these to the analysis group and found only moderate differences in socio-demographic characteristics.

These analyses are based on parent-reported tobacco smoke exposure but not on biological markers, whereas the validity is frequently criticised in relation to an underreporting of the exposure. However, a sub-study of these two birth cohorts showed a good agreement between self-reported smoking at home and measured residential air nicotine concentration as well as with cotinine measurements in children’s urine [46]. In addition, measurements of cotinine levels in children’s urine are not very helpful when longer-term effects of second hand smoke exposure will be assessed, because of the short shelf-life of approximately 24 hours. We consider a major bias of our results by the parent-reported exposure to second hand smoke not as likely. However, the effect on excess costs is less clear. If smoking is underreported, cost of children not exposed may be overestimated, resulting in an underestimation of excess costs. Furthermore, the role of the length of exposure and transitions between exposure groups could not be considered adequately and should be further investigated.

Regarding estimation of direct healthcare costs, several assumptions were necessary that may have caused over- or underestimation of costs. We replaced missing values for the number of physician and therapist visits as well as hospital days using a multiple-step single imputation technique. This may lead to smaller variances in the estimates. We did not use multiple imputations, as this did not seem feasible in our multi-step approach. We based the assessment of cost components on the suggested values published by the AG MEG [36], which were updated to the base year 2007 to account for price changes. This method has several limitations, especially regarding price variation within healthcare sectors, which is described in more detail in an earlier study [34]. As the reasons for hospital stays were not available, we used the same cost per day for all hospital stays. Actual reasons for hospitalisation might vary between groups with different exposure to smoke, which might influence the estimated cost differences. Moreover, we based cost calculation on weighted mean prices for Germany as these are assumed to reflect opportunity costs.

In infants, earlier studies found no significant association between second-hand smoking and physician visits [23,47]. Levy et al. detected no differences in children’s overall Medicaid expenditures by presence of smokers in the household [48]. One possible reason for this might be selection bias. That is, families that expose their children may also be less likely to take them to the doctor. A lower use of health services in adult smokers was found in earlier US studies [18,49]. Other studies, however, did find an association with higher utilisation of hospitals [19,22]. Yet, we found that in older children of about 10 years of age, second-hand smoke exposure is associated with a higher probability of physician visits as well as higher hospital costs. For the sum of the included cost components, the odds of positive costs, as well as the amount of costs, was significantly higher compared with children who were not exposed to smoke. Similar to our findings, van Reek et al. found increased likelihood of visiting a physician for children who were exposed to second-hand tobacco smoke 2 years before assessment [17]. In a recently published study, Adams et al. observed no significant association of maternal smoking and admission to Neonatal Intensive Care Unit but a positive effect on the length of stay of exposed infants once admitted [50], which is in line with our findings for hospital utilisation in older children. Florence et al. report higher odds for respiratory expenses in children exposed to second-hand smoke in the USA [18]. In Germany, there is a lack of evidence on the economic impact of second-hand smoke; however, Thyrian et al. estimate that tobacco smoke exposure at home is responsible for more than 14,000 hospital admissions in children. In line with our findings, they also found a longer duration of stay using a top-down approach [30]. The aim of the present study was to fill the gap based on data from a large sample of children in Germany. Cost estimates are based on a bottom-up approach. In contrast to top-down studies, which are based on aggregated data from administrative statistics, this offers the possibility of adjusting the estimates for a broader range of confounding variables. Health insurance data might allow for more precise cost estimations, but they don’t include information on parents’ smoking status. As correlation between health problems and smoke exposure is not trivial, the presented analyses are based on the excess cost approach, attempting to capture all the differences between the analysed groups of smoke exposure and minimising the need for additional assumptions. Therefore, multivariate statistical analyses did not include adjustments for comorbidities [51].

## Conclusions

This study indicates that children exposed to tobacco smoke incur higher healthcare costs. Excess costs were observed particularly for children exposed inside their homes, but also those in homes where smoking occurred on patios or balconies. These findings suggest that efforts to prevent children’s exposure to smoke should be increased considerably. As an important first step, parents could be educated not to smoke indoors at their home. However, children not exposed to tobacco smoke at all show the lowest healthcare utilisation and costs. Therefore optimal prevention should aim to help parents quit smoking completely and further increase awareness for this problem in the general population. Public policies like non-smoking rules that were lately introduced in Germany [52] as well as high cigarette taxes [53] are first steps in this direction but might not be enough. One possible approach could be to target women who quit smoking during pregnancy and prevent them from resuming to smoke. The tobacco-free initiative of the WHO could provide a framework for such efforts [54].

## Competing interests

All authors declare that they have no competing interests.

## Authors’ contributions

AB, SZ, CMT, JH and RL developed the design and analysis plan of this study. JH initiated this study and suggested the assessment of exposure to tobacco smoking. AB and SZ developed the first draft of the manuscript and performed statistical analyses. CMT ans PM revised the manuscript according to the reviewers’ suggestions including further analyses. JH, CPB, UH, SK, IL, OH, AvB, DB, UK, BS and HEW provided data from the birth cohorts. All authors (AB, SZ, JH, CMT, PM, CPB, UH, SK, IL, OH, AvB, DB, UK, BS, HEW, RL) contributed to interpretation of findings, critically reviewed each draft of the manuscript, contributed to writing and approved the final manuscript.

## Authors’ information

Helmholtz Zentrum MÃÂ¼nchen, German Research Center for Environmental Health (Ariane Batscheider, Sylwia Zakrzewska, Joachim Heinrich, Christina M. Teuner, Petra Menn, H-Erich Wichmann and Reiner Leidl) is a member of the German Center for Lung Research.

GINIplus Study Group

Helmholtz Zentrum München, German Research Center for Environmental Health, Institute of Epidemiology, Munich (Wichmann HE, Heinrich J, Sausenthaler S, Zutavern A, Chen, CM, Schnappinger M, Rzehak P); Department of Pediatrics, Marien-Hospital, Wesel (Berdel D, von Berg A, Beckmann C, Groß I); Department of Pediatrics, Ludwig Maximilians-Universität, München (Koletzko S, Reinhard D, Krauss-Etschmann S); Department of Pediatrics, Technical University, Munich (Bauer CP, Brockow I, Grübl A, Hoffmann U); IUF–Institut für Umweltmedizinische Forschung at the Heinrich-Heine-University, Düsseldorf (Krämer U, Link E, Cramer C); Centre for Allergy and Environment, Technical University, Munich (Behrendt H)

LISAplus Study Group

Helmholtz Zentrum München, German Research Center for Environmental Health, Institute of Epidemiology, Munich (Heinrich J, Wichmann HE, Sausenthaler S, Chen CM, Schnappinger M); Department of Pediatrics, Municipal Hospital “St. Georg”, Leipzig (Borte M, Diez U); Marien-Hospital Wesel, Department of Pediatrics, Wesel (von Berg A, Beckmann C, Groß I); Pediatric Practice, Bad Honnef (Schaaf B); Helmholtz Centre for Environmental Research – UFZ, Department of Environmental Immunology/Core Facility Studies, Leipzig (Lehmann I, Bauer M, Gräbsch C, Röder S, Schilde M); University of Leipzig, Institute of Hygiene and Environmental Medicine, Leipzig (Herbarth O, Dick C, Magnus J); IUF–Institut für Umweltmedizinische Forschung, Düsseldorf (Krämer U, Link E, Cramer C); Technical University Munich, Department of Pediatrics, Munich (Bauer CP, Hoffmann U); ZAUM–Center for Allergy and Environment, Technical University, Munich (Behrendt H, Grosch J, Martin F).

## Pre-publication history

The pre-publication history for this paper can be accessed here:

http://www.biomedcentral.com/1472-6963/12/344/prepub
